# A nursing care-sensitive patient satisfaction measure in older patients

**DOI:** 10.1038/s41598-023-33805-9

**Published:** 2023-05-10

**Authors:** Margarida Goes, Henrique Oliveira, Manuel Lopes, César Fonseca, Lara Pinho, Maria Marques

**Affiliations:** 1 Comprehensive Health Research Center, Universidade de Évora, Évora, Portugal; 2 Escola Superior de Enfermagem São João de Deus, Universidade de Évora, Évora, Portugal; 3 Instituto de Telecomunicações, Aveiro, Portugal; 4 Instituto Politécnico de Beja, Beja, Portugal

**Keywords:** Public health, Population screening, Health care, Geriatrics

## Abstract

As a novelty, this article proposes the empirical operationalization of an indicator sensitive to nursing care called *patient satisfaction* based on functional capacity and quality of life assessments. This was a descriptive cross-sectional study with a sample of 351 individuals aged 65 and older residing in the community. Data acquisition was performed using the structured interview method, employing a core set of 25 codes taken from the International Classification of Functioning, Disability, and Health and the WHOQOL-BREF instrument of the World Health Organization. Confirmatory factor analysis was used to infer the reliability and construct validity of the proposed model, involving three latent factors: functional capacity, quality of life, and patient satisfaction with nursing care received. The proposed model showed good reliability and construct validity, although it failed regarding discriminant validity between latent factors. The greatest statistically significant predictor of the patient satisfaction latent factor was the quality of life latent factor ($\beta =0.89;p<0.001$), followed by the functional capacity latent factor ($\beta =-0.77;p<0.001$). The findings seem to suggest that *patient satisfaction* is an indicator that may be quantitatively measurable, with functional capacity and quality of life considered very significant predictors of patient satisfaction with the nursing care experience.

## Introduction

Various international organizations/institutions, such as the World Health Organization (WHO), the United Nations (UN), the World Bank (WB), the Organization for Economic Cooperation and Development (OECD), and the European Commission (EC), among others, have turned the world’s attention to the population aging. The pace of aging has accelerated in the past decade and is expected to accelerate even faster in the next two decades. Similarly, Portugal is expected to be among the OECD countries where population aging will occur “very quickly”^1^.

Individuals are living longer, as observed by the increase in the demographic indicator *life expectancy at birth*. However, even though people live longer, these extra years of life are often unhealthy. This finding emerges by comparing the demographic indicators *life expectancy at birth* and *healthy life years at birth* (the number of years an individual is expected to live without diseases or disabilities). For example, in the European Union (EU), the number of healthy life years at birth was estimated at 65.1 for women and 64.2 for men, which represents approximately 77.5% (for women) and 81.8% (for men) of the total life expectancy at birth, according to data from EUROSTAT in 2019^2^.

Determining the healthy life years for the population aged 65 and older, whose prevalence of chronic diseases and disabilities is higher, the number of years of healthy life at age 65 (represented by the demographic indicator *healthy life years at 65*) in the EU, was estimated at 10.4 for women (6.9 for Portugal) and 10.2 for men (7.9 for Portugal), representing approximately 47.7% (for women) and 55.7% (for men) of the total life expectancy at 65 years of age (corresponding to the demographic indicator *life expectancy at 65*), according to data from EUROSTAT in 2019^2^. Portugal has a much lower position in the ranking of EU countries concerning this demographic indicator, with an estimated total life expectancy at age 65 of 30.9% and 42.7% for women and men, respectively^3–6^.

The reduction in the number of healthy life years, which significantly impacts individuals aged 65 and older, is related to the high prevalence of multiple and complex chronic diseases (multimorbidity) that cause disabilities and dependencies. A systematic analysis regarding the “Global Burden of Disease Study 2019” reported that chronic diseases accounted for nine of the top ten causes of death worldwide^7^.

A National Health Service (NHS) report showed that in 2016, 41% of the Portuguese population had multimorbidity (11% had two chronic diseases, 8% had three, and 22% had four or more chronic diseases), and 18% already reported one chronic disease. The same report found that multimorbidity increases with age and is more prevalent in women than men^8^. According to work developed by Rodrigues et al*.*^9^ in 2018, the authors reported an even higher prevalence of multimorbidity among individuals aged 65 and older, estimated at 78.3% for the Portuguese population sample used in their research (estimates reported by age groups of 65–69, 70–74, 75–79 and 80 years and older were: 72.8%, 78.2%, 81.9%, and 83.4%, respectively).

Individuals with multimorbidity need long-term care provided by multidisciplinary teams that provide integration and continuity of care. However, in most health systems in Europe, care is currently organized around specific diseases, and interventions are often accomplished to improve clinical outcomes^10^. Unfortunately, this care approach does not adequately respond to the needs of individuals suffering from multimorbidity since care focusing on managing a single disease may be impractical, irrelevant, or even harmful^10–12^. In this regard, the need for health systems to design and provide “person-centered” care aligned with the needs and preferences of the recipient has emerged^10–13^.

If “person-centered” care is a central objective of modern health systems, and health decisions are to be shared between caregivers and patients, then action is necessary to reach this goal. However, it still needs to be clarified what individuals aged 65 and older, and their caregivers value in the care they receive, and research on this topic is emerging^12^. Thus, in recent years, *patient satisfaction* has emerged as a reflection (outcome) of patients’ experience with the health care received^14^.

*Patient satisfaction* has increasingly been considered an essential indicator of the suitability and efficiency of health care delivery, making it possible to understand the extent to which such care produces effective positive changes in the individuals’ health status (suitability) and simultaneously to obtain a measure of the health care system’s performance (efficiency), i.e., an indicator that captures the suitability and efficiency of the delivery of patient-centered quality health care^14,15^.

Nursing care is one of the main components of health services^14^ because nurses represent the largest workforce in patient care^16^. Recent research has been published to assess nursing care’s effect on the recipient’s health, thus providing further visibility to nursing care. This research has added empirical evidence on nursing-sensitive indicators to measure the value of nursing care for patients^17,18^.

One of the most revealing studies on this topic was published by Dubois et al*.*^16,19^, corroborated by Rapin et al*.*^20^, and supported by Afaneh et al*.*^18^, in which the first authors developed the Nursing Care Performance Framework (NCPF) that included a matrix of indicators related to the main functions of a nursing system, sectioned into three subsystems: (1) acquiring, deploying and maintaining resources; (2) transforming resources into services; and, (3) producing changes in patients' conditions. The third subsystem of the NCPF comprises four indicators that have been summarized into the *patient satisfaction* indicator, aiming to capture the changes in a patient’s functional status, disease state, or evolving health condition according to the nursing care provided, thus covering the outcomes that reflect^16,19^: (1) patient comfort and quality of life related to care (*patient comfort and quality of life*); (2) changes in knowledge, skills, and behaviors at the patient self-care level (*patient empowerment*); (3) the patient’s functional status (*patient functional status*); (4) patient safety (*risk outcomes and safety*).

According to Dubois et al*.*^16,19^, patient satisfaction with the nursing care received is a subjective result that reflects *the interaction of their expectations of care and their perceptions of actual outcomes resulting from provider services*^16^. Although all the work related to the development of NCPF is theoretical, it is crucial to develop some empirical research related to the operationalization of this indicator, as stated by Dubois et al*.*^16,19^.

Thus, this article proposes to empirically operationalize the *patient satisfaction* indicator of the NCPF, resorting to previous research that assessed the functional capacity (developed by Goes et al*.*^21^) and quality of life (developed by Goes et al*.*^22^) of individuals aged 65 and older residing in the community. This study’s innovative nature consists of how the *patient satisfaction* indicator was operationalized, namely by using core indicators sensitive to nursing care (functional capacity and quality of life), instead of employing a single instrument specifically designed to assess patient satisfaction with the nursing care experience.

## Methods

### Study area, inclusion criteria, and sample size

This cross-sectional and descriptive study involved individuals aged 65 and older residing in the community in the south-central region of mainland Portugal, receiving nursing home care. This area, the Baixo Alentejo Region (BAR), was chosen because it is considered one of the oldest in the country (it has a large proportion of residents aged 65 and older).

The following inclusion criteria were considered: (1) being 65 years of age or older; (2) residing in BAR in their own home or the home of family or friends; (3) being interested in participating in the research; (4) being able to make their own decisions, even if they are ill or hospitalized due to the worsening of their health status; (5) having signed the written informed consent form; and (6) having answered both instruments correctly and entirely (no missing data was allowed).

The Local Health Unit of the RBA (LHUBA) database, containing 32,893 individuals aged 65 and older, was used for the sample composition^23^. The initial (random) sample included 468 individuals. However, some older adults did not answer correctly and entirely to both instruments during the interviews, resulting in missing data (117 cases). In addition, 32 older adults did not want to participate in the research, so the LHUBA health professionals did not collect their written informed consent forms. For these reasons, only 351 surveys were considered valid, fully meeting all five inclusion criteria mentioned above, and considered for analysis.

### Instruments

Two instruments were considered for data collection: (1) the Elderly Nursing Core Set (ENCS); and (2) the WHOQOL-BREF.

The ENCS was the instrument employed to assess older adults' functional capacity. It was developed initially by Fonseca et al*.* and administered to a sample of institutionalized older adults^24^. Later, it was administered to a sample of older adults residing in their homes or at family members’ or friends’ homes by Goes et al*.*^21^. It comprises 25 questions based on the International Classification of Functioning, Disability, and Health (ICF)^25^, all scored on a Likert scale ranging from 1 to 5, with higher scores indicating a higher disability level regarding his or her functional capacity. The resulting scores on a 0–100% scale yield the profile of the individual's functional capacity as follows: (1) No disability: 0–4%; (2) Mild disability: 5–24%; (3) Moderate disability: 25–49%; (4) Severe disability: 50–95%; and (5) Complete disability: 96–100%, a feature that was not used in this article^25^ (an implementation of the profiles of the individual's functional capacity was published by Goes et al*.*^22^). The list of all 25 items of the ENCS is available in Appendix A.

The WHOQOL-BREF instrument is a short version of the WHOQOL-100 quality of life assessment tool developed by the WHO^26^. It comprises 26 questions (two are of general nature and were excluded from this research since they are not linked to the quality of life domains) that measure an individual's quality of life across four domains: physical health (7 questions), psychological health (6 questions), social relationships (3 questions), and environment (8 questions), which incorporates the subjective perception of an individual’s physical and psychological health, social relationships and environment^22^. The questions ask about an individual's satisfaction with various aspects of their life, such as their physical abilities, emotional state, social support, and living conditions. The responses are rated on a Likert scale ranging from 1 to 5, with higher scores indicating better quality of life. The WHOQOL-BREF is a widely used instrument for assessing the quality of life and has already been translated into Portuguese according to the research work developed by Canavarro et al*.*^27^. The list of all 24 items of the WHOQOL-BREF can be found in Appendix B.

The ENCS and WHOQOL-BREF instruments included a header for gathering interviewees’ bio-sociodemographic data, such as age, sex, marital status, and education level.

Data collection took place from January 2016 to April 2017. Health professionals of the LHUBA^23^ conducted the interviews in the participants’ homes using both instruments simultaneously. The duration of the interviews was 45–60 min, depending on the difficulties presented by the interviewees. The interviewees were also informed that they could withdraw from the research anytime, and all data would be destroyed. The data collected by these two instruments resulted in parallel samples: functional capacity^21^ and quality of life^22^ assessments of individuals aged 65 and older.

### Data analysis

**Figure 1:**
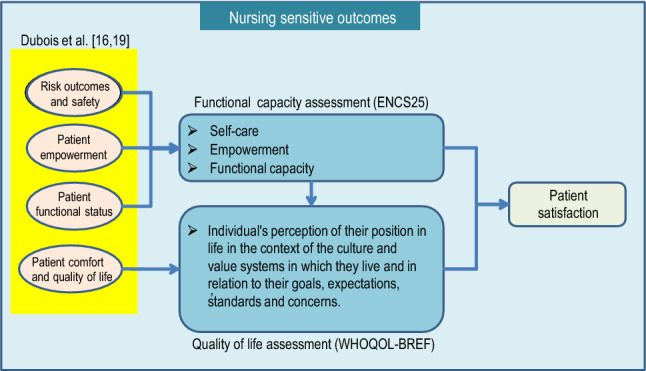
Conceptual model used in this research to operationalize the *patient satisfaction* indicator.

The conceptual model developed to operationalize the measurement of the *patient satisfaction* indicator (whose result was assigned to the output variable designated as **Sat**) is depicted in Fig. 1. The diagram shows the relationships that were established between the four dimensions that make up the “Nursing sensitive outcomes” block of the NCPF developed by Dubois et al*.*^16,19^ (highlighted by the yellow box on the left side of Fig. 1; see also Fig. 3 in Dubois et al*.*^16^, or Fig. 1 in Dubois et al*.*^19^), and the assessments of functional capacity (using the ENCS instrument^21^, whose result was assigned to the output variable designated as **Func**) and quality of life (using the WHOQOL-BREF instrument^22^, whose result was assigned to the output variable designated as **QoL**), with both constructs being represented by the two blue boxes in the central part of Fig. 1.

Subsequently, inferences were made about the association between **Func** and **QoL** output variables with the output variable **Sat**. For this purpose, the following research questions were specified: (1) does the variable **Func** manifests itself in the variable **QoL**? (2) Does the variable **Sat** manifests itself in the variable **Func**? (3) Does the variable **Sat** manifests itself in the variable **QoL**? (4) How far do the weighted scores of the three output variables vary as age increases?

The factorial validity of the models that allowed the previously mentioned research questions to be answered was confirmed by confirmatory factor analysis (CFA) using the *lavaan* package (version 0.6-11)^28^ for the R statistics software (version 4.2.0)^29^. In the *lavaan* package, the recommended method for estimating model parameters is the diagonally weighted least squares (DWLS) if ordinal data are used. This method was specifically designed when neither the assumption of normality nor the continuity property of the sampling data are considered plausible, in which the diagonal matrix of the final weights is used instead of the full weights matrix^30^.

The overall quality of fit of the CFA models was based on the following indices, as recommended by Marôco^31^: (1) ${\chi }^{2}$ statistic with correction for degrees of freedom: ${\chi }^{2}/\left(df\right)$; (2) Comparative Fit Index (CFI); (3) Tucker–Lewis Index (TLI); (4) Standardized Root Mean Square Residual (SRMR); (5) Root Mean Square Error of Approximation (RMSEA); (6) 90% confidence interval for the RMSEA population (RMSEA_CI(90%)_).

Reliability and construct validity of the CFA models were carried out based on the following procedures, as recommended by Marôco^31^: (1) individual reliability of the reflective items, by verifying whether the standardized factorial loadings ${\lambda }_{ij}$ (referred to as the *i*th reflective item of *j*th latent factor) were greater than 0.5 (that is, if ${\lambda }_{ij}\ge 0.5$); (2) construct reliability (a measure of internal consistency in scale items), by verifying if composite reliability (*CR*) of each latent factor (${CR}_{j}$ of the *j*th latent factor) is greater than 0.7 (that is, if ${CR}_{j}\ge 0.7$); (3) construct validity, according to the following steps: (3-a) factorial validity, by verifying whether the items were the reflection of the latent factors that were intended to be measured; (3-b) convergent validity, by verifying whether the average variance extracted (AVE) for each latent factor (${AVE}_{j}$ of the *j*th latent factor) was greater than 0.5 (that is, if ${AVE}_{j}\ge 0.5$); (3-c) discriminant validity, by verifying whether the expression $\left({AVE}_{l} \bigwedge { AVE}_{k}\right)\ge {\phi }_{lk}^{2}$ returns a logical value of TRUE, where ${\phi }_{lk}^{2}$ is the square of the correlation between the latent factors *l* and *k*. The factor score weights (*fsw*), inferred from the respective SEM model, were used as weights to calculate the weighted scores of the output variables **Func**, **QoL**, and **Sat**.

Finally, Spearman’s rank-order correlation (*ρ*) was the measure of association used to infer how the weighted scores of the three output variables (latent factors) **Func**, **QoL**, and **Sat** vary with the variable **Age**.

### Ethical considerations

The study protocol, design, interview procedures, research methods, and the written informed consent form were approved on July 6, 2014, by the Health Ethics Committee of the Local Health Unit of Baixo Alentejo (HECLHUBA^32^), with reference number 2/2014. The interviews only began after the respondents expressed their full agreement to participate in the research and freely signed the informed consent form. All the research methods were carried out in full accordance with the guidelines of the Helsinki Declaration^33^, aiming to protect the dignity, privacy, and freedom of the participants, as stated in the operating regulations of HECLHUBA^34^.

### Ethical approval

**Institutional review board** This research was conducted in accordance with the Helsinki Declaration and approved by the Institutional Review Board (or Ethics Committee) of HECLHUBA (protocol code 2/2014, approved in February 2014).

## Results

The sociodemographic characteristics of the interviewees are listed in Table 1. The age of the respondents ranged from 65 to 101 years, with an average of 78.1 and a standard deviation of 7.86. The sample data collected showed a higher proportion of women than men. Most interviewees were married, and 32.5% were widowed, of whom 76.3% were women and 23.7% were men. Regarding education level, approximately half of the interviewees ($46.4\%=29.6\%+16.8\%$) had no formal education, and 29.6% (57.8% women and 42.2% men) were illiterate.

**Table 1:** Sociodemographic characteristics of the sample (descriptive statistics).

Variables	N (%)
Age	
65–74	132 (37.6%)
75–84	135 (38.5%)
85 and more	84 (23.9%)
Sex	
Male	163 (46.4%)
Female	188 (53.6%)
Marital status	
Single	27 (7.7%)
Married	206 (58.7%)
Divorced	4 (1.1%)
Widowed	114 (32.5%)
Educational level	
Does not know how to read or write	104 (29.6%)
Knows how to read and write	59 (16.8%)
1st–4th grade	165 (47.0%)
More education	23 (6.6%)

**Figure 2:**
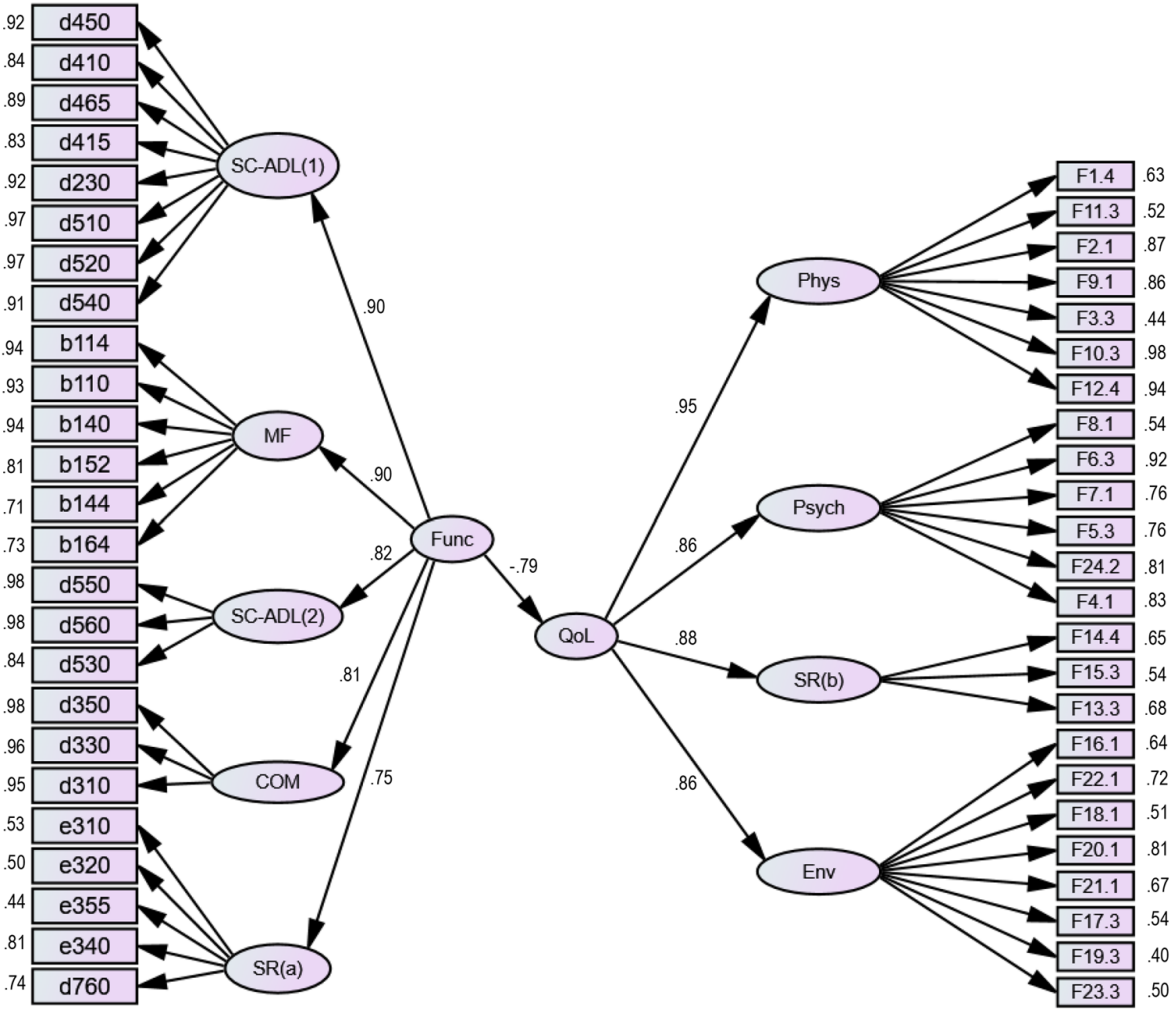
Final CFA_Func-QoL_ model that studies whether the Func latent factor manifests itself in the QoL latent factor.

Figure 2 shows the results of the CFA_Func-QoL_ model, which tests whether the **Func** latent factor manifests itself in the **QoL** latent factor, looking to answer the first research question posted in the “Data analysis” section. The 25 instruments items comprising the ENCS25, shown in Fig. 1 (left side), were grouped into five latent factors based on an exploratory factor analysis previously developed according to the research published by Goes et al*.*^21^: (1) first group of Selfcare-Activities of daily living (SC-ADL_(1)_); (2) a second group of Selfcare-Activities of daily living (SC-ADL_(2)_); (3) Mental functions (MF); (4) Communication (COM); (v) Social Relationships (SR_(a)_). Regarding the 24 items of the WHOQOL-BREF instrument, they were grouped into four latent factors, namely (see Goes et al*.*^22^ for details): (1) Physical Health (Phys); (2) Psychological (Psych); (3) Social Relationships (SR_(b)_); and (4) Environment (Env).

The CFA_Func-QoL_ model was developed without any correlation between the errors of the observed variables. The global indices revealed a very acceptable fit: (1) ${\chi }^{2}/\left(df\right)=2.488$ ($p<0.001$); (2) CFI = 0.988; (3) TLI = 0.988; (4) SRMS = 0.082; (5) RMSEA = 0.065; and (6) RMSEA_CI(90%)_ = [0.062;0.068]. The standardized factor loadings (${\lambda }_{ij}$) between the latent factors (represented by ellipses) and the observed variables (represented by small rectangles and identified by the respective code, as specified in Appendices A and B) are presented together with the observed variables for better visualization (all statistically very significant for $p<0.001$). A summary analysis of its values shows that the model presented adequate individual reliability of the reflective items (${\lambda }_{ij}\ge 0.5$ for almost all items and ${\lambda }_{ij}<0.5$ only in three cases, respectively: e355 = 0.44; F3.3 = 0.44; F19.3 = 0.40).

The CFA_Func-QoL_ model (see Fig. 2) was favorable regarding construct reliability because the latent factors mostly presented CR values greater than 0.7 (SC-ADL_(1)_ = 0.97; SC-ADL_(2)_ = 0.95; MF = 0.94; COM = 0.97; SR_(a)_ = 0.75; Phys = 0.91; Psych = 0.90; Env = 0.82; Func = 0.92; QoL = 0.94), except for SR_(b)_, whose value was 0.66 (according to Hair et al*.*^35^, values between 0.5 and 0.7 may be considered “acceptable” in the case of experimental studies). All the observed variables reflect the measured latent factor, so the model presented adequate factorial validity. Regarding convergent validity, only the SC-ADL_(1)_, SC-ADL_(2)_, and Env latent factors reported AVE values lower than 0.50, namely 0.39, 0.39, and 0.30, respectively, as shown in Table 2 (according to Hair et al*.*^35^, AVE values between 0.5 and 0.3 may be considered “acceptable” in experimental studies, as is the case of the present research). Finally, on checking the logical value of TRUE for the expression $\left({AVE}_{i} \bigwedge { AVE}_{j}\right)\ge {\phi }_{ij}^{2}$, where ${\phi }_{ij}^{2}$ is the square of the correlation between the latent factors *i* and *j*, the model failed regarding discriminant validity (see Table 2).

**Table 2:** Results regarding the discriminant validity of the CFA model presented in Fig. 2. The diagonal cells of this table represent the AVE values for each latent factor, while the values in the two lower rows (Func and QoL) show the square of the values of the correlation coefficients between factors.

	SC-ADL_(1)_	SC-ADL_(2)_	MF	COM	SR_(a)_	Phys	Psych	SR_(b)_	Env	Func	QoL
SC-ADL_(1)_	0.82	–	–	–	–	–	–	–	–	–	–
SC-ADL_(2)_	–	0.88	–	–	–	–	–	–	–	–	–
MF	–	–	0.72	–	–	–	–	–	–	–	–
COM	–	–	–	0.92	–	–	–	–	–	–	–
SR_(a)_	–	–	–	–	0.39	–	–	–	–	–	–
Phys	–	–	–	–	–	0.60	–	–	–	–	–
Psych	–	–	–	–	–	–	0.61	–	–	–	–
SR_(b)_	–	–	–	–	–	–	–	0.39	–	–	–
Env	–	–	–	–	–	–	–	–	0.37	–	–
Func	*0.80*	*0.82*	**0.66**	**0.66**	*0.56*	–	–	–	–	0.70	–
QoL	–	–	–	–	–	*0.91*	*0.74*	*0.77*	*0.75*	**0.63**	0.79

Cells filled with bold represent situations in which the expression $\left({AVE}_{i} \bigwedge { AVE}_{j}\right)\ge {\phi }_{ij}^{2}$ returned the logical value TRUE, while the italics ones indicate those that returned the logical value FALSE.

Results of the CFA_Func-QoL_ model also seem to suggest that the functional capacity was significantly manifested in the interviewees’ quality of life, with strong explanatory power because the value of the standardized regression coefficient (*β*) was − 0.791 ($p<0.001$). It should be noted that the negative value of the coefficient is because the items of ENCS and WHOQOL-BREF instruments have their response scales inverted. Given that $\beta =-0.791$ (a value qualitatively classified as “strong”) and that ${\left(-0.791\right)}^{2}=0.626$, then the **Func** latent factor explained 62.6% of the variance that occurs in the **QoL** latent factor.

**Figure 3:**
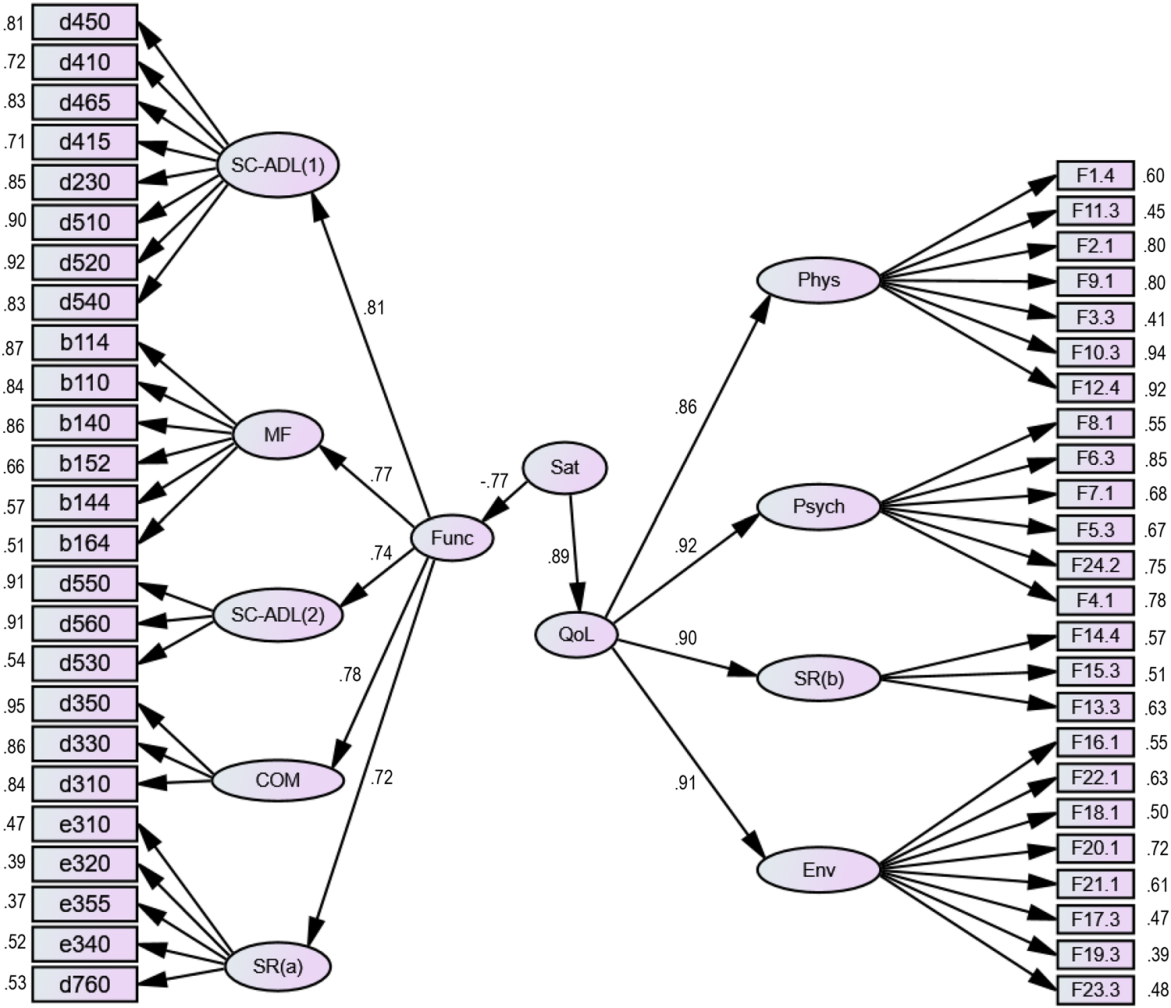
Final CFA_Func-Qol-Sat_ model that studies whether the Sat latent factor manifests itself in the Func latent factor and in the QoL latent factor.

Figure 3 shows the CFA_Func-QoL-Sat_ model that tests whether the **Sat** latent factor manifests itself in the **Func** and **QoL** latent factors, looking to answer the second and third research questions posted in the “Data analysis” section. It was also developed without any correlation between the errors of the observed variables. The model presented an acceptable global fit since ${\chi }^{2}/\left(df\right)=2.456$ ($p<0.001$). It also showed adequate individual reliability of the items because the factor loadings ${\lambda }_{ij}$ were mostly greater than 0.5 (only 18.4% were less than 0.5), and none were less than 0.3. The latter threshold of 0.3 is considered acceptable for exploratory studies^35^.

The CFA_Func-QoL-Sat_ model (see Fig. 3) showed composite reliability, with CR values consistently higher than 0.7, except for the SR_(a)_ and SR_(b)_ latent factors, which had values of 0.57 and 0.59, respectively. Concerning construct validity, the model presented adequate factorial validity because, like the previous model (see Fig. 2), the items seem to reflect the latent factors to be measured. Regarding convergent validity, the AVE values were higher than 0.5, except again for the SR_(a)_, SR_(b)_, and Env latent factors, with values of 0.21 (low value even for experimental studies^35^), 0.33, and 0.31, respectively (acceptable values in case of experimental studies^35^). On checking the logical value of TRUE for the expression $\left({AVE}_{i} \bigwedge { AVE}_{j}\right)\ge {\phi }_{ij}^{2}$, the model failed concerning discriminant validity (see Table 3).

**Table 3:** Results regarding the discriminant validity of the CFA model presented in Fig. 3. The diagonal cells of this table represent the AVE values for each latent factor, while the values in the three lower rows (Func, QoL and Sat) show the square of the values of the correlation coefficients between latent factors.

	SC-ADL_(1)_	SC-ADL_(2)_	FM	COM	SR_(a)_	Phys	Psych	SR_(b)_	Env	Func	QoL	Sat
SC-ADL_(1)_	0.68	–	–	–	–	–	–	–	–	–	–	–
SC-ADL_(2)_	–	0.65	–	–	–	–	–	–	–	–	–	–
MF	–	–	0.56	–	–	–	–	–	–	–	–	–
COM	–	–	–	0.78	–	–	–	–	–	–	–	–
SR_(a)_	–	–	–	–	0.21	–	–	–	–	–	–	–
Phys	–	–	–	–	–	0.53	–	–	–	–	–	–
Psych	–	–	–	–	–	–	0.52	–	–	–	–	–
SR_(b)_	–	–	–	–	–	–	–	0.33	–	–	–	–
Env	–	–	–	–	–	–	–	–	0.31	–	–	–
Func	*0.66*	*0.59*	**0.55**	*0.61*	*0.56*	–	–	–	–	0.58	–	–
QoL	–	–	–	–	–	*0.74*	*0.67*	*0.81*	*0.83*	-	0.76	–
Sat	–	–	–	–	–	–	–	–	–	*0.79*	**0.59**	0.69

Cells filled with bold color represent situations in which the expression $\left({AVE}_{i} \bigwedge { AVE}_{j}\right)\ge {\phi }_{ij}^{2}$ returned the logical value TRUE, while the italics ones indicate those that returned the logical value FALSE.

As shown in Fig. 3, the **Sat** latent factor manifests itself very significantly in the **Func** latent factor, with strong explanatory power because the standardized value of the regression coefficient was $\beta =-0.77$ ($p<0.001$). Moreover, the **Sat** latent factor manifests itself very significantly in the latent factor **QoL**, with strong explanatory power, because the standardized value of the regression coefficient was $\beta =0.89$ ($p<0.001$). Given that the values of the standardized beta coefficients are − 0.77 and 0.89 and that ${\left(-0.77\right)}^{2}=0.593$ and ${0.89}^{2}=0.792$, the **Sat** latent factor explained 59.3% and 79.2%, respectively, of the variance that occurs in the **Func** and **QoL** latent factors.

In short, results of the CFA_Func-QoL-Sat_ model seem to suggest that the most significant predictor of the **Sat** latent factor was the **QoL** latent factor ($\beta =0.89;p<0.001$), followed by the **Func** latent factor ($\beta =-0.77;p<0.001$).

From the CFA_Func-QoL-Sat_ model, it was possible to infer the values of the factor score weights (*fsw*), which enabled the calculation of the weighted scores of each of the three latent factors considered in this research, **Func**, **QoL**, and **Sat**. To do this, the formulation presented in Table 4 was used, which was based on the individuals’ responses to the instruments’ items and the *fsw* values (used as weights, with all being normalized to 1).

**Table 4:** The formulation used to calculate the scores of the Func, QoL, and Sat latent factors, based on the standardized *fsw* values (the respective sum for each latent factor is equal to 1) inferred from the CFA model shown in Fig. 3 and the individual responses to the instruments’ items.

Latent factors	*fsw* × items individual responses
Func	0.020 × d450 + 0.012 × d410 + 0.020 × d465 + 0.013 × d415 + 0.031 × d230 + 0.014 × d510 + 0.036 × d520 + 0.037 × d540 + 0.117 × d550 + 0.135 × d560 + 0.010 × d530 + 0.067 × b114 + 0.055 × b110 + 0.057 × b140 + 0.017 × b152 + 0.006 × b144 + 0.004 × b164 + 0.135 × d350 + 0.063 × d330 + 0.037 × d310 + 0.018 × e310 + 0.011 × e320 + 0.014 × e355 + 0.034 × e340 + 0.036 × d760
QoL	0.012 × F1.4 + 0.003 × F11.3 + 0.030 × F2.1 + 0.025 × F9.1 + 0.006 × F3.3 + 0.095 × F10.3 + 0.073 × F12.4 + 0.047 × F4.1 + 0.036 × F24.2 + 0.052 × F5.3 + 0.052 × F7.1 + 0.128 × F6.3 + 0.027 × F8.1 + 0.062 × F13.3 + 0.042 × F15.3 + 0.054 × F14.4 + 0.022 × F16.1 + 0.038 × F22.1 + 0.015 × F18.1 + 0.065 × F20.1 + 0.034 × F21.1 + 0.032 × F17.3 + 0.023 × F19.3 + 0.027 × F23.3
Sat	− 0.013 × d450 − 0.007 × d410 − 0.013 × d465 − 0.009 × d415 − 0.020 × d230 − 0.009 × d510 − 0.023 × d520 − 0.023 × d540 − 0.071 × d550 − 0.084 × d560 − 0.006 × d530 − 0.041 × b114 − 0.034 × b110 − 0.036 × b140 − 0.010 × b152 − 0.004 × b144 − 0.003 × b164 − 0.083 × d350 − 0.029 × d330 − 0.033 × d310 − 0.011 × e310 − 0.007 × e320 − 0.009 × e355 − 0.021 × e340 − 0.023 × d760 + 0.019 × F1.4 + 0.006 × F11.3 + 0.050 × F2.1 + 0.041 × F9.1 + 0.010 × F3.3 + 0.154 × F10.3 + 0.119 × F12.4 + 0.076 × F4.1 + 0.059 × F24.2 + 0.084 × F5.3 + 0.084 × F7.1 + 0.207 × F6.3 + 0.043 × F8.1 0.100 × F13.3 + 0.067 × F15.3 + 0.087 × F14.4 + 0.036 × F16.1 + 0.061 × F22.1 + 0.024 × F18.1 + 0.106 × F20.1 + 0.056 × F21.1 + 0.051 × F17.3 + 0.037 × F19.3 + 0.044 × F23.3

**Figure 4:**
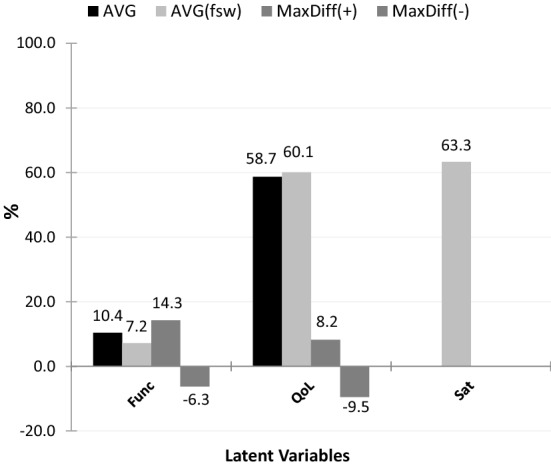
Average Func, QoL, and Sat latent factors scores, on a scale of 0–100%, using unit weights (black bars) versus employing the formulation listed in Table 4 (light gray bars). The values indicated by the dark gray bars correspond to the maximum individual differences (either positive or negative) found throughout all 351 elements of the sample.

The graph in Fig. 4 shows the average sample scores for the **Func**, **QoL**, and **Sat** latent factors: (1) unweighted averages (**AVG**—black bars), calculated using the formulation proposed by Goes et al*.*^21,22^; (2) weighted averages (**AVG**_(*fsw*)_—light gray bars), calculated employing the formulation presented in Table 4, whose weights are the *fsw* values inferred from the CFA model depicted in Fig. 3 normalized to 1. The remaining values are **MaxDiff (+)** and **MaxDiff (−)**, that is, the maximum positive difference and the maximum negative difference found throughout the 351 elements of the sample, using the expression **AVG** − **AVG**_(***fsw*)**_ (dark gray bars). For the latent factor **Sat**, the score was calculated only using the formulation shown in Table 4, as there is no reference for its calculation in the research developed by Dubois et al*.*^16,19^.

Finally, it was also tested how the scores of the three latent factors, weighted by the *fsw* values, varied with age, using the measure of association Spearman’s rank order correlation (*ρ*), looking to answer the fourth research question posted in the “Data analysis” section. The results showed the following: (1) ${\rho }_{{\varvec{F}}{\varvec{u}}{\varvec{n}}{\varvec{c}}}=0.434$ (moderate association); (2) ${\rho }_{{\varvec{Q}}{\varvec{o}}{\varvec{L}}}=-0.289$ (weak association); (3) ${\rho }_{{\varvec{S}}{\varvec{a}}{\varvec{t}}}=-0.320$ (weak association), all statistically highly significant ($p<0.001$). A brief analysis of the *ρ* values showed that the **Func** latent factor presented the highest association with variable age, whose result (${\rho }_{{\varvec{F}}{\varvec{u}}{\varvec{n}}{\varvec{c}}}=0.434$) suggests that as age increases, the likelihood of obtaining a more severe functional profile seems to increase (a finding that is consistent with the previous research conducted by Goes et al*.*^21^, when this latent factor was considered individually). Secondly, in decreasing order, a lower association occurred for the **Sat** latent factor (${\rho }_{{\varvec{S}}{\varvec{a}}{\varvec{t}}}=-0.320$), which suggests that as age increases, satisfaction with nursing care received by the interviewees seems to decrease. Finally, the third and lowest association occurred for the **QoL** latent factor (${\rho }_{{\varvec{Q}}{\varvec{o}}{\varvec{L}}}=-0.289$), which suggests that as age increases, individuals aged 65 and older seem to realize their quality of life more negatively (a finding that is also consistent with the previous research conducted by Goes et al.^36^, when this latent factor was considered individually).

## Discussion

This research proposes operationalizing the *patient satisfaction* indicator with nursing care provided to people aged 65 and older residing in the community, following the theoretical framework developed by Dubois et al*.* concerning the third subsystem of their NCPF, aimed at “*the production of outcomes that lead to positive changes in a patient’s functional status, disease state, or evolving condition as the desired end result of the interactions between patients, nursing staff, and nursing processes*”^16,19^. Rather than capturing the interviewees' perceptions of the nursing care they received and their overall satisfaction with the health care experience through an assessment based on a single instrument explicitly developed for this purpose, the proposed model was based on the assessments of their functional capacity^21^ and quality of life^22^, considered by several researchers to be priority outcome indicators sensitive to nursing care^14,17–19^. Nevertheless, the innovative nature of this research makes the comparison of the findings with those developed by other researchers somehow challenging due to the limited number of published research on the topic.

The two CFA models developed in this research performed well concerning the quality of fit, reliability, and construct validity (factorial and convergent validity). However, both failed in discriminant validity between latent factors, suggesting that functional capacity, quality of life, and patient satisfaction were latent factors that were found to be somewhat correlated, which is expected to some extent considering the type of constructs evaluated^21,22,37^.

The CFA_Func-QoL_ model developed within this research allowed simultaneously relating the interviewees’ functional capacity with their quality of life, using the ENCS^21^ and the WHOQOL-BREF^22^ instruments. With this model, it was possible to map the objective assessment of functional capacity into the subjective assessment of the interviewees’ quality of life. The findings seem to suggest a statistically significant empirical relationship between functional capacity and quality of life outcome indicators. They also suggest that when defining the nursing care needs according to the different levels of functional capacity based on a self-care model following the work developed by Goes et al*.*^21^, the respective nursing care provided seems to manifest markedly in the interviewees’ quality of life because the **Func** latent factor explains a significant portion of the variance that occurs in the **QoL** latent factor. In other words, by attempting to reduce the functional problems of the interviewees with nursing interventions, thus decreasing the score resulting from their functional capacity assessment, the nursing care provided also seems to lead to an increase in their quality of life scores. These findings make sense in theoretical terms and are aligned with those published by some researchers. As individuals age, they may experience declines in physical and cognitive abilities that can limit their ability to perform daily activities and participate in social events^38,39^. Some scientific evidence has shown that nursing care can help older adults maintain or regain their independence by assisting them with daily living activities, managing chronic health conditions (especially in individuals suffering from multimorbidity), and promoting overall health and well-being^40–42^. There is robust scientific evidence showing that nursing care contributes to improving functional capacity, helping older adults to remain active and engaged, and allowing them to live more autonomously with fewer dependencies, resulting in better physical and mental health and quality of life outcomes^40–45^.

The CFA_Func-QoL-Sat_ model developed within this research allowed relating functional capacity, quality of life, and satisfaction with nursing care provided to older adults residing in the community. The findings seem to suggest that both the functional capacity and the quality of life of the interviewees are determinants of satisfaction with the nursing care experience since the correlations obtained between the **Func** and **QoL** latent factors with the **Sat** latent factor were found to be statistically very significant. However, *patient satisfaction* with such care seems to have a more significant impact on the assessment of quality of life than the assessment of functional capacity due to a more significant proportion of the variance explained by the CFA_Func-QoL-Sat_ model regarding the former latent factor. Given that the CFA_Func-QoL-Sat_ model captured what was most similar among the three latent factors, the results suggest that *patient satisfaction* with the nursing care provided to them seems to be more related to their needs, standards, and expectations (obtained during their quality of life assessment) and not so much with the objective evaluation performed by health professionals (obtained during their functional capacity assessment). Several research studies proclaim that patient satisfaction is crucial in assessing the quality of nursing care, providing valuable insights into how patients perceive their care experiences^12,16–20^. Moreover, older adults who are satisfied with their nursing care, whose assessment is considered subjective by researchers^15,16,19,20^, seem to experience an increased quality of life^22,36,44,46^, which also results in a subjective assessment aiming at capturing older adults’ perception of their health, hopes, expectations, and feelings after the delivery of nursing care^22,44,46^, suggesting that one subjective assessment (patient satisfaction) seems to be more related to another subjective assessment (quality of life), compared to a non-subjective one (functional capacity). Finally, some researchers also report that when older adults feel that their nursing care needs are being met, they appear to feel more comfortable and secure, improving both physical and psychological well-being^14,15,20,22,46^, which seems to be aligned with the findings reported here, through the analyses of the standardized regression coefficients, namely: a decrease in the functional capacity assessment score (empowerment of interviewees' functional capacity), simultaneously seems to increase the quality of life assessment score, yielding a positive effect on patient satisfaction with nursing care provided to them.

Concerning the measures of association found between the scores of **Func**, **QoL,** and **Sat** latent factors with the variable **Age**, the results seem to suggest the following interpretations. With regard to the effect that the scores of these factors exhibit, the strongest was assigned to the **Func** latent factor when compared to **QoL** and **Sat** latent factors, suggesting that as age increases, the likelihood of older adults presenting functional problems of greater complexity seems to increase, leading to a greater need for nursing care, a finding that was already reported by Goes et al*.*^21^. As people get older, they may experience a range of physical and cognitive changes that can affect their ability to perform daily living activities, such as dressing, cooking, cleaning, and driving^21,38,39^. This decline in functional capacity can be due to various factors, including natural age-related changes in the body, the development of chronic health conditions, and environmental factors, such as living conditions and access to health care^38,39^. Thus, the association type between functional capacity and age reported in this research is aligned with that was found by other researchers, as they also reported significant evidence of functional decline or loss of independence as people age^12,38,39,47^. Regarding the measure of association between the score of **QoL** latent factor and the variable **Age**, the result suggests that as age increases, the quality of life of the interviewees seems to decrease, which is also expected and stated by some researchers, mainly due to due to health problems, financial difficulties, or social isolation^12–14,22,36,44^. However, it is important to mention that some older adults may experience an improved quality of life as they age, mainly if they can maintain their physical health through regular exercise, a healthy diet, and managing chronic health conditions properly, which is also reported in some scientific literature on the topic^47,48^. Finally, concerning the association between the score of **Sat** latent factor and the variable **Age**, the result suggests that as age increases, the satisfaction with the nursing care delivered to the interviewees seems to decrease. However, this relationship is expected because older adults will tend to be less satisfied with the impact that the worsening of functional problems may have on their quality of life, a finding that is also corroborated by other researchers^12,14,22,38,44^.

Regarding the mean scores obtained for the sample, those based on the *fsw* values inferred from the CFA_Func-QoL-Sat_ model, results seem to suggest that the interviewees are somewhat satisfied with the nursing care provided to them (average score greater than 50%), which seems to be revealing of the positive effect that such care had on their health condition. This finding reinforces the importance of providing the best possible nursing care to a patient and/or caregiver (family or friends) that effectively improves their level of rehabilitation, readaptation, and reintegration^49,50^, preferably in their homes, as the sample comprised individuals aged 65 and older residing in the community^12,13,21,38,40^. Nursing care planning based on functional capacity, quality of life, and patient satisfaction indicators seems to become more coherent with the real nursing care needs of individuals, as it makes it possible to encode a wide range of information about the patient, from which it will be possible to decide on the most appropriate nursing interventions and nursing resources to be made available^11,16,18–20^.

In summary, taking into account the strong associations found in this research between the **Func**, **QoL**, and **Sat** latent factors, the extreme relevance of the provision of nursing care to the studied population group emerges, as such care is a promoter of positive changes in their functional condition^21,38,42,45^ and their quality of life^22,44,51^, especially as age advances. In this context, the need for a person-centered nursing care setting is highlighted, as has been discussed by several researchers, ensuring the integrity and continuity of that care, making it more comprehensive and capable of effectively responding to the real needs of older adults, whose multimorbidity is more prevalent throughout their life cycle^10–12,46,49^. Measuring patient satisfaction, involving standardized assessments of functional capacity and quality of life, as a nursing-sensitive outcome indicator, provides a person-reported assessment that allows a deep understanding of that person's life, values, priorities, and preferences, all important for better management of their health condition^18–20,52^.

## Conclusions

*Patient satisfaction* seems to be a significant indicator for health care quality assessment. It is sensitive to nursing care, according to the theoretical model developed by the authors that conceived the NCPF. It is also considered in several models to be both an outcome of nursing services and a primary determinant of the overall satisfaction with the care experience. The models developed in this research and the resulting findings suggest that *patient satisfaction* indicator should be used to evaluate the contribution of the nursing profession as a reference and surplus value for the sustainability of health systems since the care provided can effectively produce changes in patients’ conditions with multimorbidity. Finally, both the functional capacity and quality of life assessments seem to be very significant predictors of patient satisfaction with nursing care.

The major limitation of this research is related to the fact that this was not a longitudinal study, so it was not possible to perform a long-term follow-up assessment, which might identify some cause-effect relationship between the delivery of nursing care and their effects on interviewees’ health condition.

## Data Availability

All data and materials in this research can be obtained by contacting the corresponding author: Henrique Oliveira (hjmo@lx.it.pt).

## Appendix A

List of ENCS items^21^.

### Selfcare-Activities of daily living—1 (8)

- d230 Carrying out daily routine
- d410 Changing basic body position
- d415 Maintaining body position
- d450 Walking
- d465 Moving around using equipment
- d510 Washing oneself
- d520 Caring for body parts
- d540 Dressing

### Selfcare-Activities of daily living—2 (3)

- d530 Toileting
- d550 Eating
- d560 Drinking

### Mental functions (6)

- b110 Consciousness functions
- b114 Orientation functions
- b140 Attention functions
- b144 Memory functions
- b152 Emotional functions
- b164 Higher-level cognitive functions

### Communication (3)

- d310 Communicating with—receiving—spoken messages
- d330 Speaking
- d350 Conversation

### Environment factors (4)

- d760 Family relationships
- e310 Immediate family
- e320 Friends
- e340 Personal care providers and personal assistants
- e355 Health professionals

## Appendix B

List of WHOQOL‑BREF items/facets^22^.

### Physical Health domain

- F1.4 Pain and discomfort
- F2.1 Energy and fatigue
- F3.3 Sleep and rest
- F9.1 Mobility
- F10.3 Activities of daily living
- F11.3 Dependence on medication or health care
- F12.4 Work capacity

### Psychological domain

- F4.1 Positive feelings
- F5.3 Thinking, learning, memory and concentration
- F6.3 Self-esteem
- F7.1 Body image and appearance
- F8.1 Negative feelings
- F24.2 Spirituality/religion and personal beliefs

### Social relationships domain

- F13.3 Personal relations
- F14.4 Practical social support
- F15.3 Sex

### Environment domain

- F16.1 Physical safety and security
- F17.3 Home environment
- F18.1 Financial resources
- F19.3 Health and social care: availability and quality
- F20.1 Opportunities to acquire new information and skills
- F21.1 Recreation and leisure
- F22.1 Physical environment (pollution/noise/traffic/climate)
- F23.3 Transport
